# Illuminating the Molecular Intricacies of Exosomes and ncRNAs in Cardiovascular Diseases: Prospective Therapeutic and Biomarker Potential

**DOI:** 10.3390/cells11223664

**Published:** 2022-11-18

**Authors:** Farheen Badrealam Khan, Shahab Uddin, Abozer Y. Elderdery, Khang Wen Goh, Long Chiau Ming, Chrismawan Ardianto, Abdul Rasheed Palakot, Irfa Anwar, Mohsina Khan, Mohammad Owais, Chih-Yang Huang, Jayasimha Rayalu Daddam, Meraj Alam Khan, Shoaib Shoaib, Md Khursheed, Sara Reshadat, Hamid Reza Khayat Kashani, Sameer Mirza, Abbas A. Khaleel, Mohammed Akli Ayoub

**Affiliations:** 1 Department of Biology, College of Science, The United Arab Emirates University, Al Ain 15551, United Arab Emirates; 2 Translational Research Institute, Academic Health System, Hamad Medical Corporation, Doha 3050, Qatar; 3 Dermatology Institute, Academic Health System, Hamad Medical Corporation, Doha 3050, Qatar; 4 Department of Clinical Laboratory Sciences, College of Applied Medical Sciences, Jouf University, Sakaka 72388, Saudi Arabia; 5 Faculty of Data Sciences and Information Technology, INTI International University, Nilai 78100, Malaysia; 6 PAPRSB Institute of Health Sciences, Universiti Brunei Darussalam, Gadong BE1410, Brunei; 7 Department of Pharmacy Practice, Faculty of Pharmacy, Universitas Airlangga, Surabaya 60115, Indonesia; 8 Department of Psychiatry, Icahn School of Medicine at Mount Sinai, New York, NY 10029, USA; 9 Interdisciplinary Biotechnology Unit, Aligarh Muslim University, Aligarh 202002, UP, India; 10 Department of Biotechnology, Asia University, Taichung 404, Taiwan; 11 Graduate Institute of Biomedical Sciences, China Medical University, Taichung 404, Taiwan; 12 Cardiovascular and Mitochondrial Related Disease Research Center, Hualien Tzu Chi Hospital, Buddhist Tzu Chi Medical Foundation, Hualien 970, Taiwan; 13 Centre of General Education, Buddhist Tzu Chi Medical Foundation, Tzu Chi University of Science and Technology, Hualien 970, Taiwan; 14 Department of Medical Research, China Medical University Hospital, China Medical University, Taichung 404, Taiwan; 15 Department of Ruminant Science, Institute of Animal Sciences, Agriculture Research Organization, Volcani Center, Rishon Lezion 7505101, Israel; 16 Program in Translational Medicine, Peter Gilgan Centre for Research and Learning, The Hospital for Sick Children & DigiBiomics Inc, Toronto, ON M51X8, Canada; 17 Department Biochemistry, Jawaharlal Nehru Medical College, Faculty of Medicine, Aligarh Muslim University, Aligarh 202002, UP, India; 18 College of Medicine, Mohammed Bin Rashid University of Medicine and Health Sciences, Dubai 505055, United Arab Emirates; 19 Department of Internal Medicine, Semnan University of Medical Sciences, Semnan 3513119111, Iran; 20 Imam Hossein Hospital, Madani Street, Tehran 1617763141, Iran; 21 Department of Chemistry, United Arab Emirates University, Al Ain 15551, United Arab Emirates; 22 Zayed Center for Health Sciences, United Arab Emirates University, Al Ain 15551, United Arab Emirates; 23 Department of Biology, College of Arts and Sciences, Khalifa University, Abu Dhabi 127788, United Arab Emirates

**Keywords:** cardiovascular disease, exosomes, ncRNA, miRNA, lncRNA, circRNA

## Abstract

Cardiovascular diseases (CVDs) are one of the leading causes of death worldwide. Accumulating evidences have highlighted the importance of exosomes and non-coding RNAs (ncRNAs) in cardiac physiology and pathology. It is in general consensus that exosomes and ncRNAs play a crucial role in the maintenance of normal cellular function; and interestingly it is envisaged that their potential as prospective therapeutic candidates and biomarkers are increasing rapidly. Considering all these aspects, this review provides a comprehensive overview of the recent understanding of exosomes and ncRNAs in CVDs. We provide a great deal of discussion regarding their role in the cardiovascular system, together with providing a glimpse of ideas regarding strategies exploited to harness their potential as a therapeutic intervention and prospective biomarker against CVDs. Thus, it could be envisaged that a thorough understanding of the intricacies related to exosomes and ncRNA would seemingly allow their full exploration and may lead clinical settings to become a reality in near future.

## 1. Introduction

Cardiovascular diseases (CVDs) represent one of the major causes of death annually and poses a serious burden to the healthcare sector of the society. The World Health Organization estimates that the number of people succumbing to CVDs may cross almost 25 million by 2030 [1]. With the advancements in healthcare systems and infrastructure, the quality of life of CVDs patients has improved substantially. Nevertheless, despite such interventions, the prevalence of heart failure (HF) still remains relatively high. As a matter of fact, cardiac tissues are composed of different types of cells which work in perfect harmony with each other owing to various delicate inter- and intra-cellular communication systems between these cells. This homeostasis is basically achieved through regulated orchestration of various signaling pathways involving autocrine, paracrine, and endocrine release of chemicals/mediators in a feedback loop system. Nevertheless, when this homeostasis is perturbed, pathological conditions are inevitable, and CVDs represent such a multifaceted phenomenon with wide range of pathologies. Accumulating evidences have highlighted the importance of exosomes and non-coding RNAs (ncRNAs) in cardiac physiology and pathology [2,3,4]. It is widely accepted that exosomes and ncRNAs play crucial role in maintenance of the normal cellular function and their potential as prospective biomarkers and therapeutic candidates are rapidly increasing. Considering all these aspects in mind, this review collates a comprehensive overview of the recent understanding of exosomes and ncRNAs in CVDs with special converge on hypertension induced cardiac complication. We provide a great deal of discussion regarding their role in cardiovascular system together with providing a glimpse of ideas regarding strategies exploited to harness their potential as therapeutic intervention and prospective biomarker against CVDs. 

### 1.1. General Introduction of Exosomes

**Figure 1:**
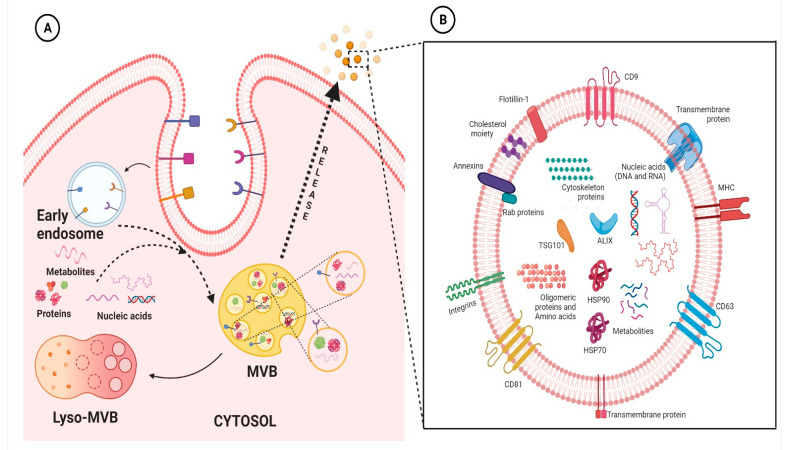
Representative figure highlighting the biogenesis of exosomes (**A**) and the typical structure of exosomes (**B**). Basically, exosome biogenesis starts with the inward vagination of the cellular membrane to form early endosomes. Thereafter, the intraluminal vesicles (ILVs) are formed, and the endosomes mature to multivesicular bodies (MVBs). MVBs fuse with the cellular membrane to release ILVs into the extracellular space, where thereafter they are denoted as exosomes. On the other hand, these MVBs can fuse with lysosomes of the cell, resulting in the degradation of ILVs (**A**). Exosomes contain various molecular entities, including nucleic acids (DNA and/or RNA), membrane anchored-proteins, cytosolic proteins, and lipids (**B**). The figures are prepared with the BioRender Software (biorender.com).

Extracellular vesicles (EVs) are membranous lipid assemblies, which carries a variety of cellular cargo including lipids, proteins, nucleic acids, metabolites, and so on [5]. Generally, these EVs are categorized based on their size and the nature of their biogenesis [6]; nevertheless, there is some overlap within this nomenclature leading to some contradiction [7]. As of yet, there are no set rules to fully categorize EVs. As a result, the International Society of Extracellular Vesicles has advocated the generic term “EVs” for the vesicles released from the cell [8]. Nevertheless, broadly speaking, there are two major classes namely microvesicles (MVs) and exosomes. MVs are also known as ectosomes, microparticles, or shedding vesicles, are vesicles having size ranging from ∼100–1000 nm and are formed from the outward budding of the plasma membrane [9,10]; whereas, exosomes are the vesicles ranging from ∼40 to 120 nm and are formed through a complex process that involves inward budding of endosomes [10,11,12]. Since the discovery of EVs, intensive research has been on-going; nevertheless, as of yet the biology of these EVs especially exosomes are not completely understood. It has been envisaged that exosomes are virtually being released from almost every cell type and they basically facilitate transport of various molecular entities, including nucleic acids, proteins, lipids, and metabolites, both locally and systemically [5,13,14,15,16,17]. Research in the frontiers of exosomes are rapidly increasing; basically a PubMed search with the keyword “exosomes” shows more than thousands of literature been published on the subject, highlighting their importance in the present scenario. Accumulating evidences have ascertained their imperative role in the context of cardiovascular physiology and pathology [18,19,20]. The origin and evolutionary perspective of exosomes and their primordial origin remains enigmatic and understanding of its plausible relation with single celled organism also remains relatively obscure. Exosomes which were once thought to be merely associated with the recycling machinery of the cell, playing role in cellular homeostasis, have undergone pragmatic shift in the field of translational medicine. They are released from wide spectrum of cells, including immune cells such as B cells, T cells, dendritic cells and stem cells, and are present in various biological fluids, such as cerebrospinal fluid, serum, saliva, urine, etc. Evidence has shown that exosomes are mechanistically and functionally diverse from its canonical counterpart and are also more heterogeneous, depending upon its origin [21]. Persistent to its endosomal origin, studies have shown the presence of major lipid rafts components consisting of ceramide, cholesterol, sphingomyelin, phosphoglycerides, long and saturated fatty-acyl chains, etc., in the exosomes. Additionally, since exosomes and multivesicular bodies (MVBs) generally originate with the aid of endosomal sorting complex required for transport (ESCRT) pathway, the proteins related to ESCRT are very prevalent and, in fact, many of them, such as HSP70, HSP90, TSG101, Alix, and tetraspanin family proteins, are considered “signature proteins” of exosomes. This, however, does not imply the absence of any other proteins since exosomes can also arise independent of classical ESCRT pathway and also it is to be noted that they act as a carrier for various protein molecules; thus, their protein profile seems to be wide and varied depending on the conditions. Recent studies have highlighted the importance of membrane proteins in the exosomes which can be leveraged to understand their origin, their preferred cellular destination and pathology of diseased state [21]. In addition to lipids and proteins, exosomes also comprise nucleic acid molecules, including mRNA, miRNA, lncRNA, circRNA, etc., as discussed below. A representative figure highlighting the biogenesis of exosomes and the typical structure of exosomes are presented in Figure 1. As a matter of fact, it is in general consensus that once these exosomes are secreted from the parent cell, they interact with the recipient/responder cells through various mechanism including clathrin-mediated endocytosis, lipid-raft mediated, and/or caveolin-mediated endocytosis, receptor-ligand mediated internalization, phagocytosis or micropinocytosis, and/or direct fusion with the plasma membrane. Lately, it has been evident that these pathways are not mutually explicit and plausibly could co-exist for the internalization of a same population of exosomes [10,22]. For example, Isabella et al., 2009 showed exosome uptake by melanoma cells through the plasma membrane fusion [23]. Similarly, another study identified exosome uptake in neurosecretory PC12 through clathrin-mediated endocytosis [24]. Perhaps, through these mechanisms, these exosomal particles modulate the activity of the recipient cells. The mechanism of exosome uptake is shown in classical cellular cargo transport physiology [10,25]. Further, it has been envisaged that the mode and level of internalization of exosomes by different cells varies widely depending on the cell type and environmental conditions. Unfortunately, but not surprisingly, it has been highlighted that the uptake of exosomes is highest in fibroblast cells and least in cardiomyocytes. Nevertheless, the underlying intricacies regulating exosomal targeting/internalization by cardiomyocyte still remains incompletely understood. Interestingly, Eguchi et al., highlighted that stem cell-derived exosomes containing the anti-apoptotic miRNA-214 are up-taken by the cardiac cells through clathrin-mediated endocytosis [26]. With paucity in the literature underlying molecular intricacies in exosomal internalization and interaction in cardiac cells, not much could be ascertained in the present scenario. Albeit certain speculations could be made based on the understanding obtained from reports on exosomes cell interaction with other cell types. It is envisaged that alteration in its profile gives plethora of information in relation to perturbation in the physiological homeostasis of the body. Interestingly, multiple lines of studies have shown that exosomes with their signature molecules plausibly act as an excellent and minimally invasive biomarker for diagnosis and prognosis of various diseases in general and CVDs in particular. To this end, much literature reviews are available highlighting the potential of exosomal signatures molecules as intriguing biomarkers for variety of pathological conditions including CVDs [27,28,29]. 

#### 1.1.1. Exosomes in Cardiac Physiology and Pathology

As a matter of fact, exosome-mediated crosstalk amongst various cell types in heart tissues have been highlighted to play crucial role in the maintenance of cardiac homeostasis, as well as in the pathogenesis of cardiac diseases [27,30]. It is well recognized that in response to various stresses, heart tissue undergoes cardiac remodeling and development of cardiac hypertrophy, apoptosis, and fibrotic responses, which eventually contribute to HF [31,32]. Albeit, understanding the molecular intricacies underlying cardiac remodeling is one of the main challenges in cardiovascular medicine. However, it has been highlighted that these responses, in part, involves vesicle-mediated cellular cross talk among cardiomyocytes and other cells in the myocardium [33,34]. Reports have shown that cardiac cells under stress have increased secretion of exosomes and the exosomal content/composition are also altered; all these aspects eventually activate or suppress various molecular signaling in the recipient cells [30,35]. Interestingly, Lyu and group have highlighted that cardiac fibroblast (CF)-derived exosomes enhanced Renin–Angiotensin System (RAS) signaling in cardiomyocytes; and it was found that attenuation of these exosome secretion considerably reversed Angiotensin II-induced cardiac injuries [36]. Similarly, researchers have highlighted that CF-derived exosomes, which were plausibly enriched with miRNAs, ensues in induction of hypertrophic responses[37]; whereas Yang et al., highlighted that exosomes derived from cardiomyocytes ensued in cardiac fibrosis through myocyte-fibroblast cross-talk [38]. Li and group has shown that plasma exosomal seemingly regulates inflammatory responses during cardiopulmonary bypass surgery through plausible involvement of miR-223 [39]. These studies explicitly highlighted the importance of exosomes in cardiac homeostasis and disease biology. In addition to playing an imperative role in maintaining cardiac homeostasis and pathophysiology; they have been highlighted to endow with potentials to revolutionize cell based therapeutic intervention against CVDs by being a potential means of cell free therapeutic strategy [40]. Accordingly, in the subsequent section, newer area into the exploration of exosomes as cell free therapeutic intervention, intriguing drug delivery platform, and novel biomarkers for CVDs had been discussed.

#### 1.1.2. Exosomes-Based Therapeutic Interventions against CVDs

Over the years, efforts have continuously been laid down to develop effective therapeutic strategies that would certainly improve the quality of the CVDs clientele. Newer therapeutic strategies are being developed, focusing not only to protect the heart tissue but also to regenerate the myocardium. To this end, accumulating evidence has highlighted the potential of stem cell therapies against CVDs; nevertheless, as of yet, these therapies refrain from showing promising results in clinical trials. Meanwhile, it has been envisaged that most of the favorable outcomes of the transplanted cells were usually indirect. Reports have highlighted that when mesenchymal stem cells (MSCs) were injected in animal model, only 6% of the injected cells were finally being retained in the infarct site [41]. It has been argued that the transplanted cells may secrete various factors/mediators, including extracellular vesicles (EVs), exosomes, growth factors, etc., that might actually play important role in mediating the beneficial effects of cell therapy. This has reinforced the holistic and emerging view of exosomes as an alternative and viable therapy. Nevertheless, despite many promising studies, the precise mechanism of exosome induced perturbations in the recipient cell still remains poorly understood. Meanwhile, taking note of other aspects, a forward leap in the arena of exosomes-based therapeutic interventions has been development of synthetic exosomes with drug delivery potentials, especially the bio-engineered targeted exosomes as detailed in the subsequent sections. Interestingly, many studies clearly indicated that exosomes in general and engineered exosomes in particular have opened newer frontier in arena of intriguing drug delivery platform and there is a high probably that these strategies may find a prosperous status in biomedical sciences in near future. 

##### (A) Exosomes as Cell-Free Therapeutic Strategies against CVDs

Owing to their various intriguing characteristics, they are increasingly being employed as a means of cell-free therapeutic interventions for myriads of obstinate diseases, including CVDs [40]. Accumulating evidence has reported that exosomes from cardiosphere-derived stem cells (CDCs) have been shown to simulate the therapeutic effects of CDCs to a large extend in animal models of heart disease [42,43,44,45]. They have been underscored to modulate cardiomyocyte hypertrophic and apoptotic responses, induce angiogenesis, and stimulate endogenous cardiomyocyte proliferation [46]. Interestingly, Zhu and the group have reported the application of human umbilical cord mesenchymal stem cell (UMSC) derived exosomes against aging related cardiac complications. In their study, the authors have ascertained that UMSC derived exosomes through the release of novel metastasis-associated lung adenocarcinoma transcript 1 (MALAT1) lncRNA suppressed aging-related cardiac complications through subsequent attenuation of NF-κB/TNF-α signaling cascade [47]. Further, it has been highlighted that exosomes produced by CDCs have been demonstrated to stimulate myocardial regeneration via transportation of miRNA to the cardiac cells [42,44,48]. In addition, Limana and group have demonstrated that exosomal from pericardial fluid considerably improved myocardial performance following myocardial infarction (MI) and has ascertained that exosomal protein clustering, an important mediator of TGF-β signaling, was plausibly responsible for the underlying cardiac protective effects [49]. Interestingly, these discoveries rationalize the use of exosomes as intriguing therapeutic intervention against CVDs.

##### (B) Bio-Engineered Exosomes as Next-Generation Therapeutic Intervention

**Figure 2:**
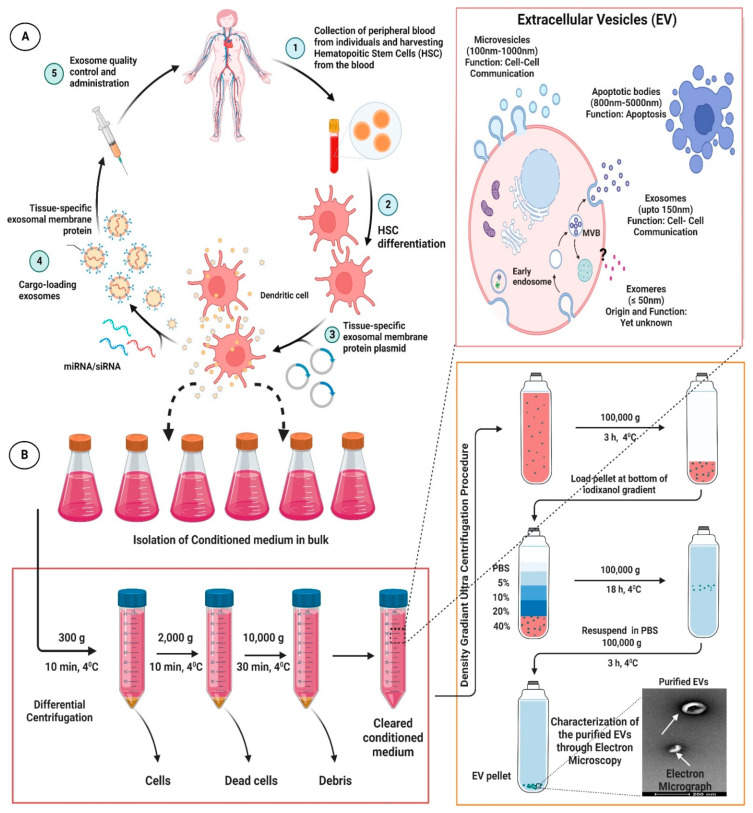
Representative figure highlighting the procedures for generation of engineered/modified exosomes for specific targeting of the therapeutic molecules to desired tissue (**A**) along with the workflow of differential ultracentrifugation for exosome isolation (**B**). The figures are prepared with the BioRender Software (biorender.com).

As a matter of fact, exosomes have been comprehended as an important cellular communication agent embodying potentials to transport diverse range of molecular entities within the biological system [50,51]. Because of their intrinsic ability to delivery molecular entities, they are considered as a promising drug delivery system (DDS) for various bioactive compounds and small molecular drugs and has been demonstrated to considerably improve their pharmacological properties against various diseases in general, and CVDs in particular. Compared with conventional drug delivery platforms, such as micelles, microemulsion, nanospheres, liposomes, and metallic nano-particulate system; exosomes offer many desirable advantages, such as lower toxicity, lower immunogenicity, high stability in circulation, better biocompatibility, and biological barrier permeability, which makes them attractive platforms for efficient delivery of therapeutic agents. Interestingly, exosomes have been used to deliver therapeutic drug and small molecules to many tissues, including the heart [52,53,54,55,56,57,58]. In fact, in recent years, engineered exosomes has been harnessed for targeted co-delivery of chemotherapeutics drug and RNA in fight against various diseases [59]. Nevertheless, exosomes in analogy with other drug delivery platforms also suffer from the drawback of endocytosis by the mononuclear phagocyte system (MPS). It has been highlighted that, when unmodified/neat exosomes were administrated systemically in animal model, they were found preferentially accumulated in the MPS organs such as liver, kidney, and spleen, which, thereafter, were rapidly cleared by bile excretion, renal filtration, and/or were phagocytized, leading to minimal accumulation of the therapeutics in the intended tissues or organs and undue delivery to un-intended tissues [60]. This bio-distribution profile and off-target effects limited the clinical acceptability of the unmodified exosomes [60,61,62]. Therefore, attempts have been made to modify exosomes for effective targeting to desired tissue. One method that has been harnessed is modification of exosomes with homing ligands or peptides, which confers them targeting capability to tissues or organs carrying the corresponding receptors. In cardiovascular system, several homing ligands/peptides are been explored for targeted therapy [52,63,64,65]. Moreover, many peptides endowed with homing potential to different cardiovascular systems, such as normal cardiomyocyte, ischemia/reperfusion injured cardiomyocytes, the vascular system etc. offers exciting avenues for exosome targeting ligands [63,64,66,67,68]. Interestingly, exosomes can be derived from an individual differentiated hematopoietic stem cells (HSC) and used for tissue-targeted cargo delivery through the expression of tissue-specific peptides. Thereafter, by loading miRNA and/or siRNA of the targeted gene, these modified tissue targeted exosomes can selectively regulate gene expression in the specific tissue corresponding to the homing peptides. Interestingly, Vandergriff et al., developed an infarct-targeting exosomes, through the use of cardiac homing peptide (CHP: CSTSMLKAC (IMTP)) to increase the efficacy and decrease the effective dose of intravenously delivered exosomes [63,64]. They basically conjugated cardiac stem cell-derived exosomes with cardiac homing peptide IMTP through a click chemistry approach using dioleoylphosphatidyl ethanolamine N-hydroxy succinimide linker. Interestingly, increased retention of the IMTP-exosomes within the ischemia/reperfusion injured heart tissues were observed to a considerable extent and improvement in cardiac function was also achieved thereof [69]. Similarly, molecular cloning and lentivirus packaging techniques were employed to engineer exosomal enriched membrane protein, i.e., Lamp2b fused with ischemic myocardium-targeting peptide IMTP. Such a fusion resulted in peptides being displayed on the surface of exosomes. Interestingly, these IMTP-exosomes displayed efficient internalization by hypoxia-injured embryonic cardiomyocyte H9c2 cells compared to blank-exosomes and subsequent increased accumulation in ischemic heart tissue were also obtained [65]. Meanwhile, attenuation of the inflammatory, apoptotic, and fibrotic responses was observed and enhanced vasculogenesis, and improved cardiac function were detected following IMTP-exosome treatment in ischemic heart. Further, Mentkowski and Lang bio-engineered a cardiomyocyte targeted exosomes that demonstrated improved cardiac retention in in vivo system [52]. Further, Mentkowski and Lang bio-engineered a cardiomyocyte targeted exosomes that demonstrated improved cardiac retention in an in vivo system [52]. To this end, the researcher selected a cardiomyocyte-specific peptide (CardioMyocyte Peptide (CMP): WLSEAGPVVTVRALRGTGS) [63,70]; which has proven ability to specifically target cardiac tissues [53,69,71,72]. The researcher ligated this CMP to the extra-exosomal N-terminus of Lamp2b. Interestingly, these cardiac-targeted CDC exosomes showed improved uptake into cardiac cells in an in vitro model; thereby leading to improved cardiac retention in in vivo system and, eventually, reduced cardiac apoptosis [52]. It has been envisaged that decorating the surfaces of the exosomes with homing ligand/entities will certainly reduce the time exosomes require to reach the therapeutic concentration in targeted tissues, and will considerably reduce the off-target effect, thereby leading to enhanced therapeutic potential. For detailed outline for the generation and isolation of the engineered exosomes; readers are advised to go through various previously published articles [52,59,65,68,69,73]. An overview of procedures for generation of engineered exosomes for specific targeting of the therapeutic molecules to desired tissue along with the workflow of differential ultracentrifugation for the isolation of the exosome are represented in Figure 2.

#### 1.1.3. Exosomes as Prospective Biomarkers for CVDs

Accumulating evidences have shown that exosomes contain diverse biological contents that plausibly is a reflection of a particular state of the system [74]. Along these lines, the vast repertoire of molecular entities that are packaged within exosomes, their versatile appearance in nearly all body fluids marks their potential candidature for prospective novel non-invasive biomarkers [75].

Amongst the exosomes content, exosomes proteins and RNA molecules especially miRNA are increasingly been reported as promising biomarkers [76]. In fact, exosomal miRNAs have been the most studied for their role as novel biomarkers for CVDs. A distinct miRNA profile has been reported by various workers in CVD patients compared to normal individuals. To this end, Matsumoto and group reported that p53-responsive circulating exosomes miRNAs viz. hsa-miR-192, hsa-miR-194 and hsa-miR-34a, were considerably upregulated in the serum of acute MI clienteles that have experienced development of HF in short period. This study highlights the importance of these exo-miRNA as plausible prognostic biomarkers for acute MI [77]. Further, studies have also shown that serum exosomal miR-9 and miR-124 levels were significantly higher in stroke patients. Concomitantly, circulating exosomal miR-9 and miR-124 might be promising biomarkers for stroke diagnosis [78]. Further, Gidlof and colleagues have demonstrated that upregulation of plasma levels of hsa-miR-208b and hsa-miR-499-5p corresponded to increase in the risk of HF, highlighting their prognostic biomarker potential [79]. 

Further, studies have envisaged the importance of various other exosomal proteins for prospective biomarkers for CVDs. To this end, Pironti et al., have reported that circulating exosomes induced by cardiac pressure overload contain functional angiotensin II type 1 receptors (AT_1_Rs); they have envisaged that the transfer of AT_1_Rs plausibly deteriorates cardiac function during blood pressure (BP) overload, thus, could help in analyzing the prognosis of the pressure overload diseased patients [80]. Similarly, the adenosine 2A receptors and dopamine receptors have also been packed within EVs and transferred to other cells, leading to an increase in BP and cardiac remodeling thereof [81]. These findings seemingly highlight for usage of these exosomal proteins as prognostic biomarkers for hypertension clienteles.

Collectively, it is reasonable to argue that many studies are being performed in basic and clinical research to understand the roles of exosomes in CVDs and to explore their prospective therapeutic, drug delivery, and biomarker potential. In parallel, it is also envisaged that albeit exosome therapy for CVDs looks promising and tempting; nevertheless, researchers in this field face numerous problems. Not only lack of thorough knowledge about cellular and molecular intricacies; but also purely technical issues as well. The issue related to low level of endocytosis in cardiomyocytes well describes the situation, and, therefore, it seems really challenging to treat CVDs or other diseases with exosomes that have not been sufficiently modified. Another problem of all the works investigating exosomes is the impossibility of isolating pure exosome preparation. Nonetheless, the research fraternities are highly optimistic and as more and more are gleaned about these aspects, it will be highly helpful in providing scope for improvisation.

### 1.2. General Introduction of Non-Coding RNAs 

It is widely accepted notion that, albeit the human genomes are transcribed into RNA; nevertheless, approximately only 2% of these transcripts have protein coding functions. Reckoning with these, researchers have started to investigate the role of ncRNAs in regulation of various physiological and pathological conditions, including CVDs. In fact, in recent years, the role of ncRNAs in cardiovascular physiology and pathophysiology has become the focus of many research endeavors [82,83,84]. It is argued that a better understanding of the involvement of ncRNAs in CVDs will offer better comprehension of the underlying intricacies which will certainly aid in novel therapeutic insights [85]. In terms of classification, basically, these ncRNAs are broadly classified based on their size; usually transcripts with nucleotide lengths < 200 nucleotides are considered as small noncoding RNAs (sncRNA); for, e.g., microRNA (miRNA), piwi-interacting RNA (piRNA), small nucleolar RNAs (snoRNAs) etc.; whereas transcripts with nucleotide lengths > 200 nucleotides are considered as long noncoding RNAs (lncRNA); for, e.g., lncRNA, which comprises of long intergenic RNA (lincRNA), enhancer RNAs (eRNAs), and sense or antisense transcripts (AS), as discussed below [86,87,88]. It is in general consensus that the cellular and temporal specificity drives the mechanism of action of ncRNAs. Basically, ncRNAs are found within the nucleus, nucleolus, cytoplasm, and even in the mitochondria. Nevertheless, extracellular ncRNAs were found outside of the cells as well. For example, ncRNA, specifically miRNA, was first observed in the plasma of the esophageal and melanoma cancer patients [89] and later on established as a potential blood biomarker for various cancer diagnoses [90]. Furthermore, ncRNAs can be transported from one cell to another through various means. For example, Valadi et al. showed that miRNA transport through exosome and they termed these RNA components as exosomal shuttle RNA [17]. Interestingly, another study found that miRNA-126 can also be transported between cells through apoptotic bodies [91]. In addition to these, ncRNAs could also be transported through carrier proteins. For example, Kasey et al., demonstrated the stable transfer of functional miRNA through high density lipoprotein (HDL) into the atherogenic mouse model [92].Interestingly, ncRNAs can be sorted and packaged into the exosome and circulate into the plasma and transported to the recipient cells [93]. Another pioneering study of plasma derived exosomal RNA profiling revealed the presence of various forms of ncRNA, including miRNA as the most abundant form in the blood circulation [94]. Horizontal transfer of ncRNAs from one cell type to another cell type has been recently established as means of intercellular communication. For example, an interesting report showed the exosome mediated miRNA transport from T-cells to the antigen presenting cells (APCs), wherein they modulate the gene expression profile of APCs [95]. Furthermore, these exosomal RNAs are involved in many pathophysiological conditions. Recently, a group showed exosomal associated lncRNAs mediated modulation of the function of l-Lacto-dehydrogenase B (LDHB), high mobility group protein 17 (HMG17) and CSF2RB which causes changes in nucleosomal architecture, and thereof enhances the cell viability [96]. These studies are attracting much attention; nevertheless, at this moment of time, it is reasonable to argue that the detailed intricacies about the exact mode of function and biology of these ncRNAs is still a matter of great interest; and with much concerted efforts from different research fraternities; more and more would be gleaned about these intricacies in near future. A representative table providing the lists of ncRNAs highlighted in various cardiac pathophysiology are depicted in Table 1.

#### 1.2.1. General Introduction of Long Non-Coding RNAs (lncRNAs)

In the recent past, studies have envisaged the importance of lncRNAs in orchestration of various cardiovascular signaling cascades [97,98,99]. As already mentioned, lncRNAs comprise a subclass of ncRNAs broadly classified as transcripts > 200 nucleotides in length with limited coding functions. Further, depending on its location in the genome and its relative distance from protein encoding genes; they can be classified as sense lncRNAs, antisense lncRNAs, bidirectional lncRNAs, intronic lncRNAs, and intergenic lncRNAs, respectively [100]. Evidence has shown that most of these lncRNAs are nuclear; however, recent studies have shown that they are also present in the cytoplasmic compartment as well. The genetic loci of lncRNAs are quite similar to that of mRNAs, but they show less coherent co-transcriptional splicing and, in addition, they predominantly possess only one intronic region. Further, in general, the expression level of lncRNAs is relatively less but more specific than normal protein coding genes, although some discrepancies can be observed in this depending on the tissue type [101]. Since the expression of lncRNAs are finely regulated, they provide vital clues regarding the developmental stages of the cell and/or disease state. Additionally, they are increasingly becoming popular for their regulatory roles in gene expression, chromatin modification, cellular differentiation besides acting as scaffolds/guides with intriguing spatial control. These functional roles are not very exclusive and many lncRNAs seem to obfuscate this notion and perform more than one function simultaneously. Interestingly but not surprisingly, the advent of newer high throughput technologies, such as RNA-seq, microarray, next-generation sequencing, and advanced transcriptomic technologies together with bioinformatic tools have heralded a new paradigm shift in our understanding of diverse functionalities of lncRNAs. Although the prevalence of such class of RNA has been known since the 1980s, there has been surge of studies showing its increasing novel regulatory functions and its role in disease progression over several decades. Interestingly, an intricate lncRNAs map was developed by Iyer et al., wherein they have characterized lncRNAs from different tissues, cancerous cells, and cell lines [102]. Similarly, Cabili et al., assembled a reference catalogue of lncRNAs from variety of body tissues and cell type [103]. This has also necessitated a comprehensive annotation resource for lncRNAs which would help researchers in better understanding of lncRNAs. These resources include GENCODE (https://www.gencodegenes.org/), LNCipedia (https://lncipedia.org/info), NONCODE (http://www.noncode.org/), TANRIC (https://www.tanric.org/), LNCat (http://biocc.hrbmu.edu.cn/LNCat/), etc. [104]. 

##### (A) LncRNAs in Cardiac Physiology and Pathology

As a matter of fact, many studies have highlighted the role of lncRNAs in regulation of CVDs; studies have envisaged the role of lncRNAs in cardiac remodeling, including cardiac hypertrophy, apoptosis, and fibrotic responses [47,105,106,107,108,109,110,111,112,113]. To this end, Zhang and group have characterized the intricacies of a lncRNA named cardiac hypertrophy-associated regulator (CHAR) in cardiac hypertrophy and delineated the underlying signaling cascade thereof [105]. Further, several studies have ascertained linkage between lncRNA and miRNA in cardiac injuries. For example, the lncRNA Plscr4 and lncRNA taurine upregulated gene 1 (TUG1) has been shown to regulate cardiac hypertrophy seemingly through regulation of miR-214 and miR-29b-3p, respectively [114,115]. Similarly, lncRNA H19/miR-675 axis has been ascertained to regulate cardiac apoptosis through suppression of VDAC1 in diabetic cardiomyopathy [116]; moreover, lnRNA myocardial infarction-regulatory factor (MIRF), i.e., lnRNA MIRF has been highlighted to promote cardiac apoptosis through regulation of the miR-26a–Bak1 axis [109]. Moreover, lncRNA ANRIL has been shown to regulate myocardial apoptosis through regulation of IL-33/ST2 pathway in acute MI animal model [117]. In addition, lncRNA NONMMUT022555, also known as pro-fibrotic lncRNA, has been reported to play intriguing role in fibrogenesis process plausibly by favoring proliferation of cardiac fibroblasts through modulation of let-7d level in MI mouse model [118]. Further, Micheletti et al., have shown that Wisp2 super enhancer associated RNA (Wisper) was associated with cardiac fibrosis and cardiac dysfunction in a murine model of MI and in aortic stenosis human patients [119]. Moreover, exosomal lncRNA AK139128 derived from cardiomyocytes under hypoxia condition has been reported to induce apoptosis and attenuate cellular proliferation in cardiac fibroblasts [120]. 

##### (B) LncRNA as Therapeutic Interventions and Biomarkers in CVDs

Meanwhile, lncRNAs have been attracting lots of attention as a potential therapeutic candidates, as well as a prospective biomarker for CVDs. A recent comprehensive study by Hu and group sheds light on the differential profile of exosomal lncRNA and mRNA in rheumatic heart disease (RHD). Interestingly, it was found that there were almost 231 lncRNA, which were differentially expressed in RHD patients in comparison to healthy clienteles. This pioneering transcriptomic analysis of the exosomal lncRNA and mRNA has provided valuable information not only for plausible biomarker for prognosis but also provided insights into intriguing therapeutic targets [121]. Further, a study by Shao et al., 2017 showed that terminal differentiation-induced ncRNA (TINCR) considerably attenuated cardiac hypertrophy through epigenetic regulation of the protein kinase CAMKII in transverse aortic constriction mouse model [122]. Previously, Micheletti and group have shown that silencing Wisper lncRNA through antisense oligonucleotide technology resulted in attenuation of cardiac dysfunction and MI-induced fibrosis in an in vivo model [119]. 

As a matter of fact, evidences have shown that lncRNAs displays dynamic alteration under pathological conditions and have long term stability in the body fluids [123]. All these features make lncRNAs as a potential non-invasive prognostic and diagnostic biomarker. To this end, Kumarswamy and group have explored the potential of lncRNAs as a prognostic biomarker for HF. Basically, the group have identified mitochondria-derived lncRNA long intergenic non-coding RNA predicting cardiac remodeling (LIPCAR), as a novel biomarker of cardiac remodeling and could certainly predicts future death in patients with HF [123]. In addition, LIPCAR was also ascertained as an intriguing biomarker for diastolic dysfunction and remodeling in type 2 diabetic clienteles [124]. Furthermore, Wang and group has revealed augmented plasma levels of LIPCAR and the paternally imprinted lncRNA H19 in clienteles with coronary artery disease (CAD) [125]. Further, Xuan et al., 2017 have ascertained the role circulating lncRNAs, i.e., non-coding repressor of NFAT (NRON) and myosin heavy-chain-associated RNA transcripts (MHRT) as novel predictive biomarkers of HF [126].

#### 1.2.2. General Introduction of Circular Non-Coding RNAs (circRNAs) 

CircRNA represents another large class of ncRNAs which as the name suggests are circular covalently closed and show spatiotemporal expression pattern in tissues and cells. They carry out their function by acting as RNA-binding proteins, sequestering agents, transcriptional regulators, as well as miRNA sponges. In addition to these, it has been reported that some selected circRNAs are converted into functional proteins as well. Despite the absence of poly-adenylation site and capping region, circRNA localizes to the cytoplasmic compartment and forms a very stable circular structure resistant to exonuclease. Albeit studies related to circRNA are accelerating; nevertheless, information regarding the function of circRNA and the ability of it to regulate the physiological and pathological conditions are relatively in infancy. In addition, most of the studies on circRNA have been carried out with limited size of cohort which results in inconclusive interpretations. Additionally, the lack of standardized procedure for evaluating circRNA has resulted in data inconsistency between different groups. These factors have limited the scope of circRNA. Nevertheless, as with lncRNA, several annotation resources for circRNA have been created which the help of researchers in better comprehension of these molecules. A repertoire of tissue specific circRNA database was created recently by Liu et al. and named generically as circRNA database (http://circnet.mbc.nctu.edu.tw/). Several other databases to further accelerate research on circRNA has also been created, such as CircRNABase (http://starbase.sysu.edu.cn/starbase2/mirCircRNA.php), circBase (http://www.circbase.org/), Circ2Traits (http://gyanxet-beta.com/circdb/), CircInteractome (https://circinteractome.nia.nih.gov/), etc. 

##### (A) CircRNA in Cardiac Physiology and Pathology

Evidence has shown that circRNA has emerged as regulatory molecule in CVDs. In addition, they are considered as novel biomarkers for CVDs, besides being considered as important therapeutic targets. With the help of high sequencing technologies, recent studies have found plenty of circRNA in heart tissues from human and mouse origin. Interestingly, heart-related circRNA (HRCR) was the pioneer circRNA, which was found to be considerably suppressed in hypertrophic heart and in HF model [127,128,129]. It has been reported that HRCR acts as a sponge for miR-223, which has been implicated in cardiac hypertrophic responses [128]. Intriguingly, it has also been shown that overexpression of HRCR could provide protection against hypertrophy plausibly through attenuation of miR-223 in a mouse model [127]. Further, whole transcriptome analysis revealed that five circRNA, namely circRNA26, circRNA261, circRNA1191, circRNA4251, and circRNA6913, were differentially expressed following cardiac hypertrophic induced by high glucose treatment. These circRNA was found to have around ~60 target miRNA for regulation [130]. Concomitantly, these differentially expressed circRNA ascertained the biologically relevant RNA markers and corresponding regulatory network in high glucose induced cardiomyopathies. Further, Wu et al., using circRNA microarray and in silico analysis ascertained that 59 plasma circRNA were differentially expressed (46 circRNAs were significantly upregulated and 13 were significantly downregulated) in human hypertensive plasma samples. Amongst these differentially expressed circRNA, has_circ_0005870 was further validated to be considerably downregulated in hypertensive clienteles [131]. In another study, researchers have highlighted the role of circRNA_000203 to promote cardiac hypertrophy plausibly through inhibition of miR-26b-5p and miR-140-3p which regulate Gata4 expression levels [132]. Further, CircRNA microarray studies have ascertain that three circRNA namely chr8:71336875j71337745, chr5:90817794j90827570, and chr6:22033342j22038870 were overexpressed in case of rat coronary artery endothelial cells (CAEC) treated with TGF-β1 [133]. This study interestingly highlighted the potential role of differentially expressed circRNAs during TGF-β1-related CVDs.

##### (B) CircRNA as Therapeutic Interventions and Biomarkers against CVDs

Further, recent studies have exploited circRNA as therapeutic interventions against CVDs. Interestingly, in the case of atherosclerosis, circANRIL has been demonstrated to bestow athero-protection through modulation of ribosomal RNA (rRNA) maturation and governing pathways related to atherogenesis [134]. Similarly, circRNA_010567 was shown to ameliorate MI through attenuation of TGF-β1 [135]. Likewise, a promising study by Zeng et al. evaluated the potential of circRNA circ_Amotl1, which is highly expressed in neonatal cardiac tissue and manifests cardio-protective functions by binding to PDK1 and AKT1 [136].

As already mentioned, linear RNA molecules have been highlighted as potential biomarkers [137]; to this end, as circRNAs are more stable and resistant to exonucleases compared to linear RNAs, as a result it bestows more advantageous properties for its potential to act as a biomarker [138]. Thus, it is highly reasonable to argue that circRNAs are more superior to its analogous mRNAs and lncRNAs as prospective biomarker candidates in terms of abundance, stability, and specificity. In analogy with lncRNAs, Zhao and group have envisaged the potential of peripheral blood circular RNA hsa_circ_0124644 as a diagnostic biomarker of CAD [139]. A recent meta-analysis of several databases have demonstrated that two circRNA namely circCDKN2BAS and circMACF1 have prospective potentials to be used as circulating biomarker in CVDs [140]. Furthermore, a study in 2017 highlighted the usage of the circRNA, myocardial infarction associated circular RNA (MICRA) to predict the risk in MI clienteles [141]. Further, the circRNA HRCR described above could also be potentially considered for biomarker repository [138,142]. Likewise, hsa-circ-0005870 described above might represent a novel diagnostic biomarker for hypertension [131].

Collectively, the aspects that they are abundant, stable, as well as evolutionally conserved in tissues, saliva, exosomes, and blood offers enormous potential to extend the current landscape of prognostic and diagnostic biomarkers for CVDs, as well as for other diseases [123,143]. However, it is a matter of great interest that amongst exosomes, ncRNA, and exosomal ncRNA, which one would show better candidature as prospective biomarkers for CVDs is still not known.

Taken together, although each molecular entities viz. exosomes and ncRNA have distinctive role in cardiovascular system; nevertheless, the importance of cross talks between these molecular entities as regulator of various events in cardiovascular system should not be overlooked at the same time [144].

**Table 1:** Representative table providing the lists of ncRNAs highlighted in various cardiac pathophysiology.

RNA	Disease	Mechanism/Functions	References
***LncRNA***			
TINCR	Cardiac Hypertrophy	Silencing of CaMKII protein kinase	[122]
Plscr4	Cardiac Hypertrophy/ Heart Failure	Down-regulation of miR-214 expression	[114]
TUG1	Cardiac Hypertrophy	Down-regulation of miR-29b-3p	[115]
CHRF	Cardiac Hypertrophy	Down-regulation of miR-489	[106]
MIAT	Cardiac Hypertrophy	Regulation of TLR4 expression by sponging miR-93 in cardiomyocytes	[107]
FTX	Cardiac Apoptosis	Modulation of Bcl212 expression; Inhibition of miR-29b-1-5p	[108]
MIRF	Cardiac Apoptosis	Inhibition of miR-26a	[109]
ANRIL	Cardiac Apoptosis	Regulation of IL33/ST2	[117]
H19	Cardiac Apostosis/ Cardiac Fibrosis	Reduction of VDAC-1; Increment in collagen and TGF-β levels, reduction of Dus5 expression	[116,125]
NONMMUT022555	Cardiac Fibrosis	Reduction in level of let-7d	[118]
Wisper	Cardiac Fibrosis	Regulation of expression of a profibrotic form of lysyl hydroxylase 2	[119]
(GAS5)	Cardiac Fibrosis	Inhibition of miR-21; Modulation of endothelial cells via exosomes and macrophage apoptosis	[110,111]
Mhrt	Cardiac Fibrosis	Interaction with the chromatin-remodeling factor Brg1	[126]
SRA1	Cardiac Fibrosis	Inhibition of miR-148b	[112]
MEG3	Myocardial infarction	Regulation miR-183 level	[113]
AK139128	Myocardial infarction	Regulation of cellular activities of cardiac fibroblasts in vitro and in vivo	[120]
MALTA1	Aging induced cardiac dysfunction	Inhibition of NF-κB/TNF-α signaling pathway	[47]
***CircRNA***			
HRCR	Cardiac Hypertrophy/ Heart Failure	Acts as sponge for miR-223	[127,128,129]
CircRNA_0005870	Hypertension	-	[131]
CircRNA_000203	Cardiac Hypertrophy	Inhibition of miR-26b-5p and miR-140-3p which regulate Gata4 expression levels	[132]
CircANRIL	Artherosclerosis	Regulation of ribosomal RNA (rRNA) maturation and modulation of pathways related to atherogenesis	[134]
CircRNA_010567	Myocardial Infarction	Attenuation of TGF-β1	[135]
CircRNA_Amotl1	Doxorubicin induced cardiomyopathy	Binding to PDK1 and AKT1	[136]

## 2. Conclusions

Since the discovery of exosomes and ncRNAs, they have garnered much attention across the research fraternities; nevertheless, their intricacies, especially in relation with CVDs, are not completely understood. Nonetheless, in recent years, research in these fields has expanded greatly. It is argued that as the challenges in the field are gradually addressed, it will be highly instrumental to better understand the underlying intricacies regarding their biology and function, especially in CVDs. However, there are still various daunting challenges that are important stumbling blocks to truly harness their potential in clinical settings. These includes establishment of optimal dose and route of administration, better understanding of the immunogenicity of these molecular entities upon administration to the model animals, improved understanding of their pharmacokinetics and pharmacodynamic parameters, development/optimization of tools to comprehensively characterize them, etc. At this moment of time, it is reasonable to argue that these challenges need to be addressed on an urgent basis. Accordingly, a better understanding of these intricacies, along with addressing the underlying challenges will provide a fundamental basis for improving their efficacy for improved therapeutic intervention to efficiently deal with not only CVDs but also other debilitating diseases as well with equal potency.
